# Black Youths’ Perspectives: Importance of Family and Caregiver Involvement in the Mentor–Mentee Relationship

**DOI:** 10.3390/healthcare10112181

**Published:** 2022-10-31

**Authors:** Lakindra Mitchell Dove

**Affiliations:** School of Social Work, Portland State University, P.O. Box 751, Portland, OR 97207, USA; lakindra@pdx.edu

**Keywords:** black adolescents, youth mentoring, positive youth development, family engagement, community care

## Abstract

Research shows that mentorship can significantly influence the lives of youth. As a society we are becoming more diverse and aware that cultural needs for youth of color are more complex. We have seen an increase in formal mentoring programs that offer services to Black youth. As this shift continues, it is imperative that culturally responsive services are considered. Little research exists that explores the importance of family engagement within the mentor–mentee relationship. When working with Black youth, it is important to understand cultural practices found within the Black family that could have an influence on the mentor–mentee relationship. This study uses qualitative inquiry to explore the perspectives of 12 Black adolescents, ages 14–18, participating in a youth mentoring program. Through individual interviews, the participants described their experiences and observations of their mentor’s engagement with their family/caregiver. Findings suggest that Black youth value a genuine connection between their mentor and family/caregiver, they often assign a familial role to their mentor, and they adhere to cultural practices observed within the Black family. These results have implications for culturally responsive practices for youth mentoring programs to incorporate when working with Black youth and families.

## 1. Introduction

Over the past few decades, there has been a shift in the demographics of youth participating in formal mentoring programs. In a national study on youth mentoring programs, Garringer et al. [1] found that approximately 76% of youth participants identified as youth of color. This shift has included more engagement with youth from marginalized and racial/ethnic backgrounds, which has prompted discussion on how to best support these youth. However, there has not been a substantial shift in the foundational structure and philosophy of mentoring, thus not accounting for the cultural needs of youth participating in these programs [2]. For example, if youth serving organizations are not aware of the particular needs and/or challenges of a specific population or community and attempt to provide traditional services that may focus solely on academic or prosocial outcomes, it may result in the organization not integrating other services, supports, or programming that could affirm the cultural needs of youth. The existing literature speaks to the benefits of a mentoring relationship for youth [3,4,5]. These benefits include positive outcomes in academic, behavioral, and social domains [3,6]. Research shows that mentorship can significantly shape the lives of youth. However, as society becomes more diverse and cultural needs more complex, it is important to understand how mentorship may differ for marginalized youth. Many community-based mentoring programs serve youth who reside in impoverished, economically disadvantaged, and under-resourced communities, and are often youth of color [7]. In terms of Black youth, this is a population that has seen an increase in formal mentoring programs within their communities. As we seek greater knowledge and understanding of the field of youth mentorship, it is critical to pay attention to Black youth as frequent users of youth services, and to ensure that services are rendered in a culturally responsive manner that supports the cultural needs of Black youth. In this paper, Black refers to individuals who reside in the U.S. and identify as Black/African American.

### 1.1. Adolescence, Culture, and Youth Mentoring

The period of adolescence is a vital phase for identity development [8]. In addition to navigating this developmental period, Black youth also encounter the reality of historical oppression and what it means to identify with a particular race or ethnicity that has consistently been subjected to oppression, discrimination, and injustice in society [9]. In their review of the current literature that addresses the importance of culturally specific mentorship programs, Sanchez et al. [7] suggest that these types of programs may have a positive impact on racial and ethnic identity development among youth of color. This speaks to the importance of the need for youth serving programs to incorporate materials that support the cultural needs of these youth. It is also important to emphasize the role of culture and how cultural differences may impact how relationships are formed and whether a relationship is perceived as positive and supportive. Sanchez et al. [10] explored how cultural mistrust may be a barrier for youth of color in forming meaningful relationships with White mentors or mentors of a different racial/ethnic background. This is also important to note when working with the family/caregiver of mentees, as gaining their trust can be vital to the sustainability of the mentor–mentee relationship [11]. Some studies have found that the lack of an understanding of cultural nuances contributed to failed mentor–mentee relationships [12]. The youth mentorship literature discusses the importance of culture and family engagement, but few studies have explored these factors from the youths’ perspective. Additionally, the literature reveals a significant gap in research that explores racial/ethnic identity development and cultural influences within the mentor–mentee relationship. This study seeks to explore the experiences of Black youth within the mentor–mentee relationship.

### 1.2. Racial Socialization

Racial socialization is an essential component of positive youth development, specifically when working with Black youth. This includes protective factors identified by researchers as critical to supporting a healthy racial/ethnic identity, such as racial identity, racial socialization, and Africentric worldview [13,14,15]. It is also a critical component to consider within the mentor–mentee relationship and researchers suggest that highlighting race/ethnicity may contribute to the quality of the mentor–mentee relationship [7]. There are various ideas about what constitutes racial socialization. For the purpose of this paper, we will focus on communication and modeling within racial socialization. Lesane-Brown [16] suggests that there is a difference between focusing on messages of cultural pride and racial barriers, noting that both types have been found to have a positive impact on youth development. Grills et al. [17] note that Africentric cultural orientation and racial socialization could be considered protective factors for youth who encounter racially charged and stressful situations. They found that a strong Africentric cultural orientation contributes to healthy youth development. When considering the cultural nuances of the Black family and the potential influence on the mentor–mentee relationship, especially when mentors may be considered an extension of the family unit, it is imperative for mentors to be aware of racial socialization processes that can be used as tools to support healthy youth development and family engagement.

### 1.3. Family Engagement in Youth Mentoring

This study focuses on the experiences of Black youth engaged in professional mentoring relationships, in which mentors are paid for their on-going relationship with youth. Few studies have examined the impacts of professional mentorship [18,19,20]. Of those few, findings are limited regarding the professional mentors’ engagement with mentees’ family/caregiver. Salazar et al. [20] conducted a study that included professional mentors, teachers, caseworkers, and caregivers of children involved in the child welfare system. They found that caregivers appreciated the support of the professional mentor in navigating complex systems, connecting with services and supports, and building connections among providers and stakeholders in the families’ lives. Participants also emphasized the importance of consistency and continuity, related to the model of support for children and caregivers. Additionally, professional mentors and caregivers both highlighted the importance of the relationship between the mentors and caregivers, with caregivers describing mentors as a part of the family, and mentors providing emotional support to caregivers. These findings provide some context for the potential benefits of professional mentorship as it relates to family engagement. It is also important to examine professional mentoring relationships within the context of culture and the Black family, particularly when these relationships are intended to develop over an extended period of time. In the youth mentoring literature, there has been an increase in research that theoretically and empirically examines family involvement in the mentor–mentee relationship [11,21,22]. Additionally, researchers have examined protective factors within mentoring relationships that support Black youth [17]. They have also explored the role of immediate and extended family members, noting the positive influence they can have on Black youth development [23,24,25]. Within the youth mentoring literature, these relationships are referred to as natural mentors. Given the cultural socialization processes of the Black family, which are often consistent with a communal versus individualistic approach, exploring the nature of family/caregiver engagement in youth mentoring is imperative.

The formal mentoring literature emphasizes the consideration of parental influence on youth mentoring relationships [11,21]. A study conducted by Spencer et al. [11] included the perspectives of parents of youth involved in a community-based youth mentoring relationship and found that parents were active participants in their child’s mentor–mentee relationship, embracing roles such as collaborator, coach, or mediator. They also found that parents who were able to establish a relationship with the mentor, felt more comfortable with their child’s mentor–mentee relationship. Some studies that include findings from the perspectives of mentors and youth mentoring programs, suggest that mentors do not actively engage parents in the mentoring process [26] or that mentors prioritize their relationship with the mentee and attempt to maintain distance in their relationship with the families [27]. Although recent best practices for youth mentoring programs suggest incorporating family engagement [28], some mentors and programs have yet to fully integrate this practice. This raises concern as this approach may contribute to an oversight of various cultural practices that influence the mentoring process. Additionally, there is little to no research that explores family/caregiver engagement from the youth’s perspective of the mentor’s relationship with the family/caregiver.

## 2. Methods

This study was developed in the context of exploring the role of racial and ethnic identity development within the mentor–mentee relationship. This paper discusses themes that developed during conversations with youth that centered the nature of the relationship between their family/caregiver and mentor, which prompted further inquiry regarding the influence of the parent/caregiver’s role on the mentor–mentee relationship. The goal is to highlight the experiences of Black youth regarding family/caregiver engagement in the mentor–mentee relationship.

### 2.1. Participants

The study included twelve participants who self-identified as Black, African American, or mixed race (including Black). Four identified as female, 7 identified as male, and 1 identified as non-binary. Three participants were in middle school (8th grade) and 9 participants were in high school or recent graduates. The youth participants ranged in age from 14–18. All of the participants reported having worked with their current mentor for at least 1 year, with the exception of 1 participant. Mentors who participated in the study were recruited based on having youth who identify as Black on their caseload. Four mentors participated. Three identified as female and one identified as male. A total of 16 participants, including youth and mentors, were interviewed.

### 2.2. Setting

The study included youth and mentors who are involved in a chapter located in the Pacific Northwest, of Friends of the Children (FOTC), a youth serving organization that includes a national network of independent nonprofit organizations. The main goal of the program is to employ “professional” mentors to provide long-term mentoring to youth who may reside in underserved or under-resourced communities, are in need of additional support (academic/social/emotional) or could benefit from adult support. In collaboration with local schools, youth are identified in kindergarten or first grade for consideration to participate in the program through a 6-week direct observation process, which occurs in the school in collaboration with school staff. The FOTC commitment is to provide a continuous mentor for youth who remain in the service area for 12 years, typically through high school graduation.

### 2.3. Procedure

Individual interviews were conducted remotely via zoom in accordance with the IRB protocol. Due to the University’s research regulations as a result of COVID, no face-to-face research activities were permitted. To minimize the risk of coercion into participation, the alumni coordinator who did not have a pre-established relationship with youth or their legal guardians, made the initial contact to youth and the legal guardian of youth who met the study’s criteria to determine if youth were interested in participating. Mentors were asked to share a flyer containing information regarding the research study, with youth on their caseload. Once interested youth were identified, the researcher followed up with the legal guardian to discuss the study and obtain consent. Youth assent was verbally obtained prior to beginning the interview. Written research materials were provided in hard copy and electronic formats.

Using a semi-structured interview guide, participants were asked questions in the following areas: matching process for mentor–mentee, mentor–mentee relationship status, understanding of racial and ethnic identity, experiences navigating adolescent identity development, support from mentor in navigating identity development, and discussions with mentor regarding experiences of racism, discrimination, prejudice, or oppression. Youth participants completed a basic demographic questionnaire, including age, racial/ethnic background, gender, school, and the racial/ethnic background and gender identity of their current mentor. Similar demographic information was gathered from mentor participants. Permission to audio record the interview was requested from each participant and all interviews were transcribed verbatim by a third-party service. Each interview was approximately an hour long and participants received $40 for their participation in the study. Data collection occurred from August 2021 to June 2022.

### 2.4. Researcher Positionality

The researcher of the study identifies as an African American woman who has historical knowledge of the area where the study occurred and the communities that participants are a member of. This knowledge may have facilitated participants’ comfort in sharing information and experiences, especially in regard to sensitive topics such as identity development and experiences of oppression, racism, and discrimination. Based on the researcher’s historical knowledge and prior connection with the area/communities, the researcher was mindful to not make assumptions based on the researcher’s experiences and was intentional about asking participants to explicitly discuss/explain their experiences to help prevent bias in interpretation and analysis of data. The researcher invited participants to provide their own definition and understanding of cultural terms throughout the interview process. The researcher took notes during interviews about specific comments, observations, and researcher’s reactions, in addition to writing memos following each interview. Given the researcher’s familiarity with the cultural nuances and complexities of various communities, the analysis was influenced by insider knowledge of aspects of the participants’ experiences that may not have otherwise been acknowledged.

### 2.5. Analysis

The interviews were transcribed, and each transcript was read in its entirety multiple times to construct a summary of initial themes across interviews. Braun and Clarke’s reflexive thematic analysis approach [29] was used to analyze the data. The process was initiated by first reviewing the data, then open coding was used, which is integral to theme development and allows the researcher to use the data to identify meaning [29]. Initial codes were identified by the researcher and themes were constructed based on patterns within the data. Morgan [30] suggests the use of quotations as evidence for researchers to support their interpretations. The findings are organized based on themes from the data, with participants’ “quotes to illustrate meaning” of the themes. The researcher was involved in all aspects of analysis and was the sole coder.

## 3. Results

Two primary themes were identified relating to the mentees’ perceptions of the importance of family/caregiver involvement within the mentor–mentee relationship. These themes include (1) Mentor connection with parent/caregiver; (2) Familial role of mentor, and subthemes of the impact of the pandemic on the mentor–mentee relationship and the impact of mentor loss. The first theme highlights the importance of the mentor’s relationship with the mentee’s parent/caregiver. In most cases mentees referenced their primary caregiver. The second theme highlights familial roles assigned to mentors by mentees and addresses discussions across participants about how mentors developed relationships with a mentee’s siblings, who were not a part of the program, but at times included in events and outings as a member of the family unit. The primary theme identified among the mentors was the relationship with the primary caregiver of the mentee. Mentors discussed their approach to engagement and experiences working with the family/caregiver.

### 3.1. Mentor Connection with Family/Caregiver

The first primary theme was the mentor connection with the parent/caregiver. One mentee explained the difference in engagement between one mentor who she was able to establish a strong relationship with and other mentors she had throughout the program. She discussed the effort that this mentor made to also establish a relationship with her mother, given her mother is a single parent. She discussed the struggles her mother encountered and how beneficial the mentor was in supporting her mother. This relationship proved to be very beneficial later when the family encountered a stressful experience that included involvement with the justice system. The mentee articulated how the mentor made a difference illustrated by this statement:

“I feel like with my other mentors it was just about me, but with [mentor] she made an effort because she knew that I’m being raised in a single-parent household and it’s kind of hard for my mom. And so, she made sure that she created a bond with my mom. She made sure to check on [mom] and see if she needed anything, to see how she was doing. My mom went through a rough time [during court proceedings] and had to take off from work. And [mentor] made efforts to hang out with me and my mom, take us to the movies. So, it was really important that she had a bond with me and my mom, not like the other mentors did necessarily.”

When asked to further describe what was different about her experience with this mentor compared to other mentors, the mentee explained:

“I feel like [mentor] was just very different. She took a different approach than everyone else. It kind of felt like everyone else was just doing their role, job title, and [mentor] did that and more. She made it as if this is what she genuinely wanted to do and this is not something she’s getting paid for.”

In this case, although the mentee reported having approximately four to five mentors during her time in the program, it was helpful for her to have had various experiences with mentors to assess what felt meaningful to her in the ideal mentor–mentee relationship, which extended beyond the one-on-one relationship with her to include her mother.

Another mentee discussed how the pandemic impacted his relationship with his mentor, as he was becoming closer to him during the time, they were able to spend together. He noted, “I would treat him like family, just because he’s like family, my white family. I respect him a lot.” He discussed his experience with having more than one mentor while in the program, and his observation that it appeared that mentors were not allowed to meet family and that he would try to introduce his mentors to all of his family. He noted the importance of his current mentor’s willingness to be around his brothers, sharing information and having the opportunity to discuss information with his brothers, and having his brothers hold him accountable as well. He indicated that his mentor was open to allowing family members to engage in activities. He also discussed the complexity of his family dynamics and how his father was incarcerated for most of his childhood. He explained:

“Having a mentor really takes your mind off that type of stuff… I don’t need a dad to really show me what somebody else could teach me… once he [father] got out of jail, he couldn’t really teach me anything, couldn’t tell me anything… I always got more game from a mentor, somebody who is not my family than somebody in my family.”

In this experience, the mentee was able to depend on the mentor as if he were an extended family member, which seemed to also have a positive impact on the mentee’s relationship with his brothers, trusting the information shared by the mentor and helping his brothers engage in conversations and be mindful of their actions.

Another mentee who has had the same mentor since kindergarten and is now in the eleventh grade, noted how her mentor helped connect her mom to resources to pay bills. She described how this occurred on various occasions. She also explained that her mentor has been mindful of including her mother around the holidays, emphasizing “she just cares about my family in general”.

These illustrations suggest that youth were able to observe and appreciate the connection between their mentor and primary caregiver, similar to familiar relationships that are often established within a family unit. Mentees used descriptors such as genuine, open, and close to explain the relationship between their mentor and parent/caregiver. These excerpts provide great insight into how familial practices within the Black family also show up in the mentor–mentee relationship with Black youth, particularly the emphasis on respect for the mentor based on their relationship with their primary parent/caregiver.

### 3.2. Familial Role of Mentor

Another primary theme was the familial embrace of mentors extending beyond the role of the mentor. Throughout the interviews, many participants used terms of endearment to refer to their mentor and describe their relationship. Some of the mentees even used familial terms, referring to their mentors as a second mom or big sister. One mentee stated, “he was basically like a dad to me, a dad/older brother.” Another mentee described his relationship with a former mentor as “my first older best friend.” He also explained his relationship with his current mentor and stated:

“We’ll talk about some uncomfortable stuff that I wouldn’t talk about with my brothers because they would look at me like I’m weird, expressing myself, so I feel like I’m expressing myself to [mentor] like he’s my best friend before I was able to express myself to cousins”.

In this excerpt, the mentee emphasized the nature of the mentoring relationship by demonstrating how he prioritized sharing information with his mentor before his brothers or cousins. This also alludes to the level of trust that exists within the mentor–mentee relationship, to the extent that the mentee felt comfortable being vulnerable and sharing sensitive information.

Another mentee described in detail how her mentor has been there for her and her family. She emphasized the importance of family and when asked what role she would assign her mentor, she replied “that mom.” This mentee has been with the same mentor since starting the program, which also suggests the benefit of a consistent, long-standing relationship.

### 3.3. The Impact of the Pandemic on the Mentor–Mentee Relationship

One notable finding is the discussion of how mentors maintained their relationships with mentees during the pandemic. This topic naturally surfaced for some mentees when asked about engagement in activities and things that they did with their mentor. For some mentees, the beginning of the pandemic was a time when they were able to make a clear distinction in their relationship with their mentor due to how their mentor maintained the relationship. Some mentees talked about genuine efforts to reach out and stay connected. Other mentees emphasized gestures such as weekly food baskets, written notes, and consistent communication with their family. One mentee stated:

“He used to drop off these little food boxes to us when COVID first started. That was thoughtful of him. There’s certain stuff that he’d do, but he didn’t have to, and he would go out of his way to do. He’s a respectable dude.”

Additionally, mentors maintained a connection virtually by playing video games, watching tv shows, and cooking. To some extent the actions of the mentors during the pandemic solidified their relationship with their mentee and their role within the family unit. When asked to elaborate on how her mentor was checking on her more, one mentee shared:

“… when they would come deliver snacks during COVID, they would stay a little longer, have conversations about what’s going on and see if we needed anything… just reassuring us that we have FOTC and they have our backs.”

Some mentees realized that for their mentor, this was more than a job, and expressed appreciation for how their mentor checked-in on them and their family during very challenging and stressful times.

### 3.4. The Impact of Mentor Loss

Another notable finding is the mention of change in mentors, particularly when the change occurred abruptly and without much explanation. Some mentees seemed to speculate why this occurred, with a common statement such as ‘I think they got another job.’ Other mentees emphasized the sudden change especially when they had been working with the mentor for a period of time and had a good relationship established. In some instances, mentees talked about their hesitancy in working with another mentor, and when they did, how it took them longer to open up and establish a relationship.

### 3.5. Relationship with the Primary Caregiver

The main theme for the mentors was the relationship with the primary caregiver. In discussing their approach to family engagement, all four mentors discussed the importance of establishing a relationship with the primary caregiver. They also reported having extensive youth mentoring experience, within and outside of the program. One mentor had only been with the program for about 1½ years, while other mentors had 5–12 years with the program. When discussing their engagement practices, two mentors mentioned learning through experience the importance of engaging the family/caregiver first. One mentor stated:

“The first year is really getting to know them and getting comfortable with each other, making sure that the youth and their family, their guardians, that’s a huge piece, are comfortable with you… that first year I think, showing the guardians respect and consistency.”

Another mentor explained the importance of honoring and respecting the guardian’s parenting style and practice and being open to learning about the family’s culture and values. She discussed how throughout her experiences, she has learned to ask a lot of questions and clarify things with guardians. A practice that she finds really important. “I think my approach changed in just including the guardians more and not making assumptions and knowing that the way I parent or my lived experiences aren’t universal.” The other mentor discussed how he shifted his practice after attempting to engage the youth first with activities and the mother would decline. He realized that he needed to hang out and get to know the youth in his environment first, before taking him out to do activities, emphasizing the importance of, at first, “staying close to home, building a relationship with the families, the caregivers”. He also mentioned the importance of community connection and investment, “If you’re hiring folks who are from the community, who are invested in the community, they’ll stay longer because they care… They’re [other mentors] not from these communities so they’re not as invested from my perspective. “Utilizing community resources such as local organizations and community centers seemed to be another engagement strategy for this mentor.

Another mentor discussed her experience trying to engage a family who previously worked with a mentor who had not established a rapport with the family. She noted, “I showed up at the house every day for four months, dropped off care packages, books. I was like, alright, I ain’t going nowhere. I’m just showing up every day.” She emphasized the importance of gaining the trust of the family, which is something she tried to convey in her actions. Similarly, another mentor stated, “... you don’t have access unless you understand the parents, unless the parents feel respected by you.” Both mentors illustrate the importance of respect conveyed to the family/caregiver and how they engage the family/caregiver. One mentor mentioned how her approach to working with parents has changed over the years. She noted that she is more inclusive of guardians and less likely to make assumptions about parenting styles or lived experiences.

## 4. Discussion

The findings from this study emphasize the importance of family/caregiver engagement in the mentor–mentee relationship in the context of professional mentorship. The mentees revealed that mentors were able to cultivate meaningful relationships with the parent/caregiver to foster a positive relationship between the mentor, mentee, and family unit. There was not any variation in responses based on the gender of the mentee or the race/ethnicity of the mentor. Overall, mentees emphasized the quality of the relationship. In describing their observations of the interactions of mentors with their family/caregiver, many mentees seemed to note the following characteristics of these relationships: genuine, open, and close. These characteristics may be of value when considering ways in which mentors could enhance their relationship with the family/caregiver, as they suggest the type and quality of a relationship that may be important to youth to see modeled by their mentor and family/caregiver. Mentees mentioned the impact of an abrupt ending on the mentor–mentee relationship. Some mentees talked about the loss of a mentor, especially if that mentor had been involved in their life for a significant period of time and had established a meaningful relationship with their family. Oftentimes the mentees were not sure why the mentor left and did not have an opportunity to say goodbye. The manner in which they described these experiences was akin to a disruption to the family unit. One mentee indicated how his mentor left and that was it. Some mentees were aware of a transition to a new position within the organization or with another organization. This highlights the importance of youth mentoring organizations establishing a process for closure when abrupt or unplanned endings occur, especially for professional mentors who establish long-term relationships with youth and families. This support could be beneficial if the youth transitions to working with another mentor. It is also a way to model healthy endings in relationships.

The reference to mentors as a family member or best friend is consistent with familial associations within Black culture, where individuals who are respected and considered close to the family become fictive kin. Billingsley et al. [25] define fictive kin as “individuals who are unrelated by birth or marriage but take on family-like roles” (p. 794). Findings from this study are consistent with the literature on natural familial mentoring relationships among Black youth. Billingsley et al. [25] found that when primary caregivers had more really close relationships with adult relatives, their children also reported more really close relationships with adult relatives. They also noted that by modeling closeness and allowing interactions among their children and adult relatives, primary caregivers both directly and indirectly facilitated these relationships. The study also addressed youth agency in creating and maintaining intergenerational bonds. In this study mentees discussed various types of relationships with their mentors and it appeared that their observations of their mentors’ interactions with their family/caregiver significantly impacted the quality of the mentor–mentee relationship. This suggests that Black youth, whether consciously or unconsciously, continue to engage in a cultural practice of familial role assignment, similar to what typically occurs within the family system.

This research also illuminates the need for culturally responsive tools, strategies, and practices that youth mentoring programs employ to support mentors who serve Black youth. It also highlights the importance of the relationship that organizations have with communities where services are provided. The organization should be embedded within the communities and ensure that mentors understand the nuances of communities. This was also noted by a mentor who discussed his investment in the community and hiring practices of the organization. When considering a community care approach to mentoring, it is vital that mentors are aware of community resources and support that can be instrumental to the mentee’s development. In this way, a mentor serves as a guide, connecting to and circling back to the community as a source of support. From a cultural perspective, this aspect of communal care is inherent in how children are looked after and cared for. For youth mentoring organizations that choose to serve youth from these communities, it is imperative that they do not operate in silos, and understand the function of social networks within youth mentoring relationships as discussed by Keller and Blakeslee [31]. This approach can be detrimental, especially when considering the holistic needs of youth. It is also important that they expand beyond traditional target goals, focusing on a specific area of development, such as social and emotional skills, or risk aversion, such as teenage pregnancy or contact/involvement with the juvenile justice system. Black youth need to experience their communities in healthy, positive, and supportive ways, and learn how to access resources that exist. This approach strengthens ties to the community so when the mentor or organization is no longer involved in the mentee’s life, the mentee is able to depend on their community. These resources are not always directly connected to access to funds. A community can be well resourced in culturally specific ways to support youth development.

It should be noted that all youth participants were actively engaged with the program and reported having a positive relationship with their current mentor. This study did not capture the perspective of youth who did not have a favorable relationship with their current mentor or may have disengaged from the program. The youth who agreed to participate in this study may have been motivated by their positive experiences and felt more comfortable talking about these experiences. Additionally, this study did not seek to explicitly explore the role of family/caregiver engagement in the mentor–mentee relationship. Future studies that intentionally inquire about this role could contribute to the body of knowledge regarding the influence of familial and cultural practices on the mentor–mentee relationship, the benefits and drawbacks of family engagement, and how youth mentoring organizations can incorporate culturally responsive and inclusive familial practices into the mentoring process.

### Implications for Practice

In the context of professional mentorship, which often includes an extended period of involvement with mentees and their families, it is important to understand family dynamics and cultural nuances. Similar to the roles that parents/caregivers played in the mentor–mentee relationship outlined in the study conducted by Spencer et al. [11], mentees notice the benefits of mentors having an established relationship with their family/caregiver. This is a crucial component for mentors to consider during the initial phase of family engagement. During this time, it also may be beneficial to ask questions regarding family culture and assess the value of family/caregiver involvement in the mentor–mentee relationship. Although some mentees and their family/caregiver may not prefer significant involvement in the relationship, or the amount of engagement may vary, it is important for mentors to know their preference and be able to navigate a relationship with the family/caregiver. It is also important for mentors to broaden their scope of practice to consider the benefits of the community and a community care approach, especially when working with families who may access various supports and resources and youth who may be connected with other support programs. Additionally, it is helpful for mentors to understand how the community may be impacting the youth/family and the ways in which the community can be a resource. Youth mentoring programs and organizations could benefit from the results of this study by examining their philosophical approach to family engagement. The findings suggest that when working with Black youth, a model that incorporates a communal approach is one that seems to be culturally responsive, especially in the context of family values. Similarly, Albright et al. [32] emphasize the importance of applying a social justice lens in mentor–mentee relationships and ensuring that current intervention models address inequalities, marginalization, and processes of oppression that exist. Culturally relevant family system frameworks should be considered to support the relationship among the mentee, mentor, and family unit. These are some initial suggestions for mentors and youth mentoring organizations to take into consideration when exploring ways to enhance their service delivery for Black youth and families.

## 5. Conclusions

The findings from this study are reassuring in that youth participants recognize the importance of family/caregiver engagement in the mentor–mentee relationship. The experiences shared by youth could be a starting point to further explore policies and practices and implications for training, strengthening mentor–mentee relationships, and enhancing the importance of culture and family/caregiver involvement in youth mentoring. There seems to be a philosophical shift occurring within the youth mentoring field, as the literature continues to support the engagement of youth within the context of their family and community, moving away from a traditional one-on-one model to one that is inclusive and collaborative, and acknowledges the powerful role of family and community in the lives of Black youth. 

## Data Availability

The data presented in this study are available on request from the corresponding author. The data are not publicly available due to privacy.
